# Kinetics and Mechanistic Studies on the Reaction between Cytochrome c and Tea Catechins

**DOI:** 10.3390/antiox3030559

**Published:** 2014-08-19

**Authors:** Lihua Wang, Elizabeth Santos, Desiree Schenk, Montserrat Rabago-Smith

**Affiliations:** Department of Chemistry and Biochemistry, Kettering University, 1700 University Ave, Flint, MI 48504, USA; E-Mails: lwang@kettering.edu (L.W.); santose2@chemistry.msu.edu (E.S.); desi.schenk@gmail.com (D.S.)

**Keywords:** cytochrome c, catechins, kinetics, mechanistic studies, antioxidant, chemo-preventive

## Abstract

Green tea is characterized by the presence of an abundance of polyphenolic compounds, also known as catechins, including epicatechin (EC), epigallocatechin (EGC), epicatechin gallate (EGC) and epigallocatechin gallate (EGCG). In addition to being a popular beverage, tea consumption has been suggested as a mean of chemoprevention. However, its mode of action is unclear. It was discovered that tea catechins can react with cytochrome c. When oxidized cytochrome c was mixed with catechins commonly found in green tea under non-steady-state conditions, a reduction of cytochrome c was observed. The reaction rate of the catechins was dependent on the pH and the nature of the catechin. The pseudo-first order rate constant obtained increased in the order of EC < ECG < EGC < EGCG, which is consistent with previously reported superoxide reduction activities and Cu^2+^ reduction activities of tea catechins.

## 1. Introduction

**Figure 1:**
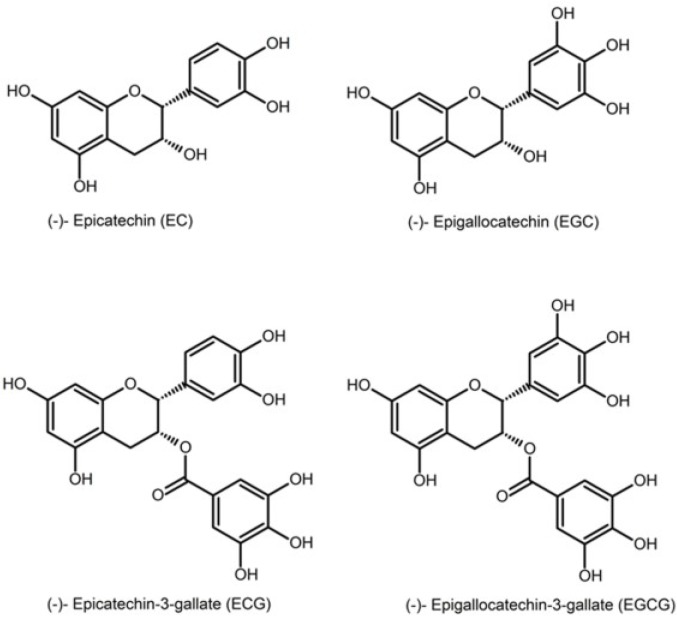
Different catechins found in tea.

Tea, brewed from the plant, *Camellia sinensis*, is one of the most consumed beverages in the world, consumed by two-thirds of the world’s population [1]. Green tea is produced by steaming freshly harvested leaves, yielding a dry, stable product [1]. It is characterized by the presence of an abundance of polyphenolic compounds, also known as catechins. Catechins contain a benzopyran skeleton with a “phenyl group substituted at the 2-position and a hydroxyl (or ester) function at the 3-position” [1]. The major catechins found in green tea are epicatechin (EC), epigallocatechin (EGC), epicatechin gallate (EGC) and epigallocatechin gallate (EGCG). EGCG is the most abundant and the most biologically active compound [2] (Figure 1: structures of the major catechins found in green tea). Green tea contains 30%–42% catechins by dry weight; it is the main source of natural antioxidants.

In addition to being a popular beverage, tea has been used for medicinal purposes, Tea consumption appears to decrease cancer risk and cardiovascular diseases, but its mechanism of action is uncertain [3,4]. Studies have shown that green tea and polyphenol compounds extracted from green tea have anticarcinogenic activity in animal models [5,6]. The proposed cancer prevention mechanisms include protection from oxidative stress, inhibition of enzymes, inhibition of growth factor-related cell signaling and the promotion of apoptosis in cancer cells [7,8].

The signal transduction pathways affected include the upstream of activator protein-1 (AP-1) and nuclear factor kappa B (NF-κB), activated through proline-rich kinase signaling. These transcription factors are known to be significant in tumor promoter-induced cell transformation and tumor promotion [9].

Other “studies show that many anti-cancer drugs induce the apoptosis of cancer cells and that catechin compounds induce apoptosis” [10] without affecting normal cells [1]. Additionally, it has been determined that the extent of their apoptosis-inducing effect is dependent on their polyphenolic structure.

The most commonly discussed mechanism for the biological activity of catechins involves their anti-oxidative activities. Reactive oxygen species induce oxidative stress, which has been linked to tumor promotion in many tissues. These reactive oxygen species include superoxide, hydroxyl, hydroperoxyl, peroxyl and alkoxyl radicals. Reactive nitrogen species include nitric oxide and the peroxynitrite anion. Catechins have the ability to prevent the formation of reactive oxygen and nitrogen species, which play pertinent roles in carcinogenesis [11]. Moreover, flavonoids are known to directly scavenge the superoxide anion and peroxynitrite. Recently, green tea extracts have been shown to be effective in scavenging reactive oxygen species when incorporated into plastic films for food packaging [12].

It has been reported that the main site of catechin antioxidant action depends on the structure of the catechins [13]. Computational studies predict that their activity comes from the B-rings and ortho-diphenols [14,15].

Cytochrome c is a small heme protein, important in mitochondrial respiration [16]. It is an important protein in the biological electron transport chains, assisting in the transport of electrons from organic substrates to oxygen. In addition, it has been discovered recently that it also plays an important role in the process of apoptosis. Cytochrome c is a metalloenzyme that requires a heme cofactor. In the electron transport chain, mitochondrial cytochrome c transports electrons from complex III to IV. The small, approximately 100-amino acid protein is conserved across most species. When the protein is released from the mitochondria into the cytoplasm, it has been found to initiate apoptosis in various cells with the addition of various compounds. Flavonoids were used to induce apoptosis via cytochrome c in leukemia cells. EGCG was found to do the same in bile duct cancer cells. Cytochrome c injections into the cytoplasm of neuronal cells also induced apoptosis after nerve cell growth factor deprivation.

We have discovered a new reaction between cytochrome c and catechins. Because of the importance of cytochrome c in apoptosis, understanding the interaction of tea components with cytochrome c would provide insights that may help in elucidating the mechanisms of the health benefits of green tea. This paper discusses the results of the kinetic and mechanistic study of the reaction between cytochrome c and various tea catechins.

## 2. Experimental Section

### 2.1. Materials and Equipment

Tea catechins, (−)-epicatechin (EC), (−)-epigallocatechin (ECG) and (−)-epigallocatechin gallate (EGCG), bovine heart cytochrome c, potassium phosphate and D_2_O (99%) were purchased from Sigma-Aldrich (St. Louis, MO, USA and used without further purification. The kinetic studies were performed using a Cary 100 UV/Vis spectrophotometer (Palo Alto, CA, USA)).

### 2.2. Kinetic Study

The reactions were performed under anaerobic conditions using 10 or 20 μM of cytochrome c and an excess amount of a tea catechin (EC, ECG, EGC or EGCG). The concentration of the catechin was at least ten times that of the concentration of cytochrome c. A 0.3-M potassium phosphate buffer was used to maintain the pH at 7.0 or 7.7 at 25 °C. The reactant solutions were purged with nitrogen before being mixed by injection into a sealed cuvette. The progress of the reaction was monitored at 550 nm using a Cary 100 UV/Vis Spectrometer for a time period of four half-lives.

The kinetic studies on the presence of BHT (butylate hydroxytoluene) were performed following the same protocol; except that 0.10 or 0.25 molar equivalent of BHT *vs.* EC was mixed with EC, and then the mixture was reacted with cytochrome c. The buffer solution used for the BHT studies contained 10% ethanol, due to the low solubility of BHT in water.

A preliminary ^1^H-NMR study was carried out by recording the proton NMR spectra every 10 min under anaerobic conditions using a 400-MHz Varian NMR. The solution contained cytochrome c (0.8 mm) and 0.5 equivalents of (–)-epicatechin in deuterated water.

## 3. Results and Discussion

**Figure 2:**
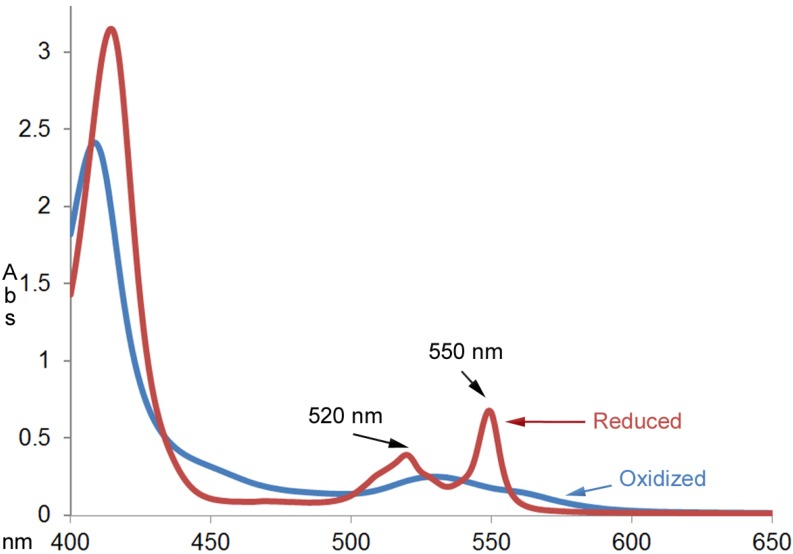
UV-Vis absorption spectra of cytochrome c (0.025 mm) before and after reacting with excess amount of EGCG (1.00 mm) for an hour at room temperature.

When oxidized cytochrome c was mixed with catechins commonly found in green tea (EC, ECG and EGCG) under non-steady-state conditions, the reduction of cytochrome c was observed. Figure 2 shows the absorption spectra of cytochrome c (0.025 mm) before and after reacting with an excess amount of EGCG (1.0 mm) for an hour at room temperature. The UV-Vis absorption spectrum of the product was characteristic of the reduced cytochrome c with the calculated millimolar absorptivity of 26.9 mM^–1^ cm^–1^ at 550 nm. The millimolar absorptivity value was close to the value of 28.0 mM^–1^ cm^–1^ reported by the product specification sheet from Sigma (St. Louis, MO, USA)).

The kinetics of the reaction between cytochrome c and the tea catechins was studied as described in the Experimental Section.

**Figure 3:**
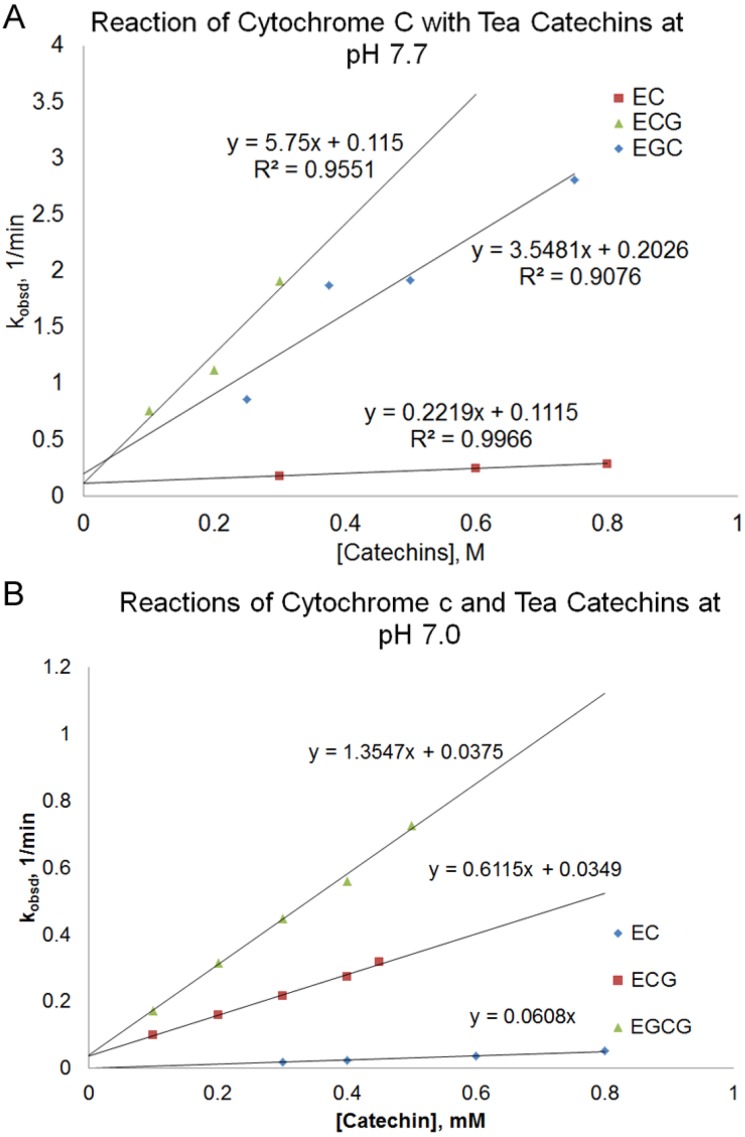
Plot of pseudo-first order rate constant, k_obsd_, *vs.* the concentration of the catechin for the reaction of different green tea catechins with cytochrome c at pH 7.7 (**A**) and pH 7.0 (**B**).

The pseudo-first order rate constant (k_obsd_) of the reaction depended on the nature and the concentration of the tea catechin used and the reaction pH. At pH 7.7, there was a linear relationship between the pseudo-first order rate constant (k_obsd_) and the concentration of the catechin used. (Figure 3A, Equation (1)).

*k_obsd_* = *k*_1_ + *k*_2_[*catechin*] (1)

According to Figure 3A, the slope of the line, k_2_, increased in the order of EC < EGC < ECG < EGCG. The reaction between EGCG and cytochrome c at pH 7.7 was too fast to be measured using a conventional UV-Vis spectrophotometer. There is a small *y*-intercept (k_1_) that was similar for different catechins.

In order to obtain measurable rates for the reactions between EGCG and cytochrome c, the reaction was also studied at pH 7.0 for some of the catechins (Figure 3B). The reaction rate of the catechins at pH 7.0 was slower than those obtained at pH 7.7. The order of increasing k_2_ values, EC < ECG < EGCG, at pH 7.0 was consistent with the order observed at pH 7.7. For ECG and EGCG, the relationship of k_obsd_ *vs.* [catechin] was consistent with Equation (1) at pH 7.0, with similar k_1_ values for both catechins. However, the k_1_ value for EC was determined to be zero. Because there are two terms in Equation 1 (term_1_ = k_1_ [cytochrome c] and term_2_ = k_2_ [catechin] [cytochrome c]), there should be two terms in the rate law for the reaction between cytochrome c and green tea catechins; Equation (2):

*Rate* = *k_obsd_*[*cyt.c*] = *k*_1_[*cyt.c*] + *k*_2_[*catechin*][*cyt.c*] (2)

This indicates that there are two pathways for the reaction: a catechin-dependent pathway, in which the rate determining step is a second order reaction between cytochrome c and the catechins, and a catechin-independent pathway, in which the rate determining step is independent of the concentration of the catechin.

The catechin-dependent pathway can be described as follows: 

Step 1 (rate determining step): electron transfer reaction between the oxidized cytochrome c and catechin to form the reduced cytochrome c (cyt.c) and a catechin free radical (catechin·):

*cyt.c*(*ox*) + *catechin* → *cyt.c*(*red*) + *catechin* · (3)

Step 2: electron transfer reaction between another oxidized cytochrome c and the catechin free radical to form a reduced cytochrome c and a two-electron oxidized catechin:

*cyt.c*(*ox*) + *catechin* · → *cyt.c*(*red*) + *catechin*(*ox*) (4)

Or two catechin free radicals react with each other to form a dimer:


2*catechin* · → (*catechin_ox_*)_2_ (5)

**Figure 4:**
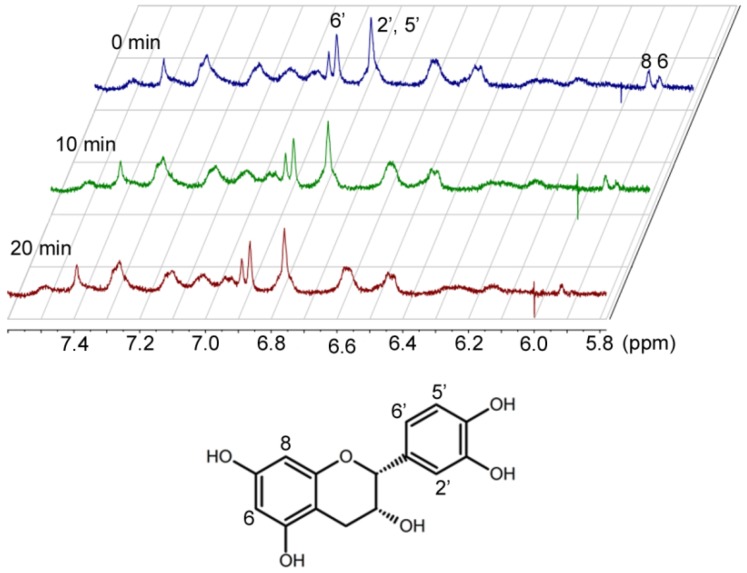
Proton NMR spectra of epicatechin and cytochrome c over time.

Kinetic data alone cannot differentiate the two possibilities for Step 2. These two alternatives can only been distinguished by the structural characterization of the oxidized catechins. Preliminary ^1^H-NMR studies of EC and cytochrome c over time indicated that hydrogen 6 of EC [17] disappears (Figure 4). Characterization of the reaction products is currently underway in our laboratory.

The rate of the catechin-independent pathway did not change with the concentration of the catechin. The possibility that this pathway involves the reduction of cytochrome c by a component in the solvent was ruled out, because, in the absence of the tea catechin, the reduction of cytochrome c did not occur. The proposed mechanism for the catechin-independent pathway consists of cytochrome c undergoing a slow transformation to a different form. The conformational change is the rate determining step of the pathway. The transformed cytochrome c then reacts with the catechin. Therefore, even though the catechin participates in the reaction, the rate law is independent of its concentration, because it is involved in a step after the rate-determining step. The catechin-independent pathway is described as follows: 

Step 1 (rate determining step): structural transformation of cytochrome c:

*cyt.c*(*ox*) → *cyt.c*(*ox*)* (6)

Step 2: electron transfer between the transformed cyt.c (ox)* and catechin to form the reduced cytochrome c and a catechin free radical:

*cyt.c*(*ox*) * +*catechin* → *cyt.c*(*red*) + *catechin*· (7)

The rest of the pathway should be the same as the catechin-dependent pathway, but it involves the transformed cytochrome c.

The fact that k_1_ values were independent of the nature of the catechins except for EC at pH 7.0 supports the proposed mechanism (Figure 2B). The reason that EC and cytochrome c at pH 7.0 did not show the catechin-independent pathway is probably because the electron transfer reaction between EC and transformed cytochrome c at pH 7.0 is slower than the conformational change of cytochrome c. Thus, the rate determining steps in both pathways involve EC, which leads to a rate law that appears to have one pathway that is first order in EC and first order in cytochrome c.

As it can be seen from Figure 1, for reactions carried out using the same concentration of different types of tea catechins, the pseudo-first order rate constant obtained increased in the order of EC < ECG < EGC < EGCG. This is consistent with previously reported superoxide reduction activities and the Cu^2+^ reduction activities of tea catechins (Table 1) [18].

**Table 1:** A comparison of the relative reactivities of green tea catechins towards different oxidizing agents.

Catechins	Superoxide Reduction IC_25_ (μm) [15]	Reduction of Cu^2+^ RC_25_ (μm) [15]	Cytochrome C Reduction at pH 7.7 k_2_ (mm^−1^ min)
EC	20.6	218	0.222
EGC	3.22	38.4	3.55
ECG	2.29	24.3	5.75
EGCG	1.45	12.5	Too fast to measure

Finally, the rate of the reaction was faster at pH 7.7 than at pH 7.0. This is because at pH 7.7, some of the catechins (EGCG (pK_a1_ = 7.75 [19]), ECG (pK_a1_ = 7.76 [19]), EGC (pK_a1_ = 7.73 [19]), EC (pK_a1_ = 8.72 [20])) are partially deprotonated, which results in a partial negative charge on the molecule and a stronger electron donating ability. This may facilitate the electron transfer process between the catechins and cytochrome c. The result is consistent with the pH-dependent radical scavenging capability of green tea catechins reported previously [16]. Cytochrome c is located in the intermembrane space of the mitochondria, which has a pH of 6.88 [21]. Therefore, the kinetic results at pH 7.0 are relevant to the environment of cytochrome c *in vivo*.

**Figure 5:**
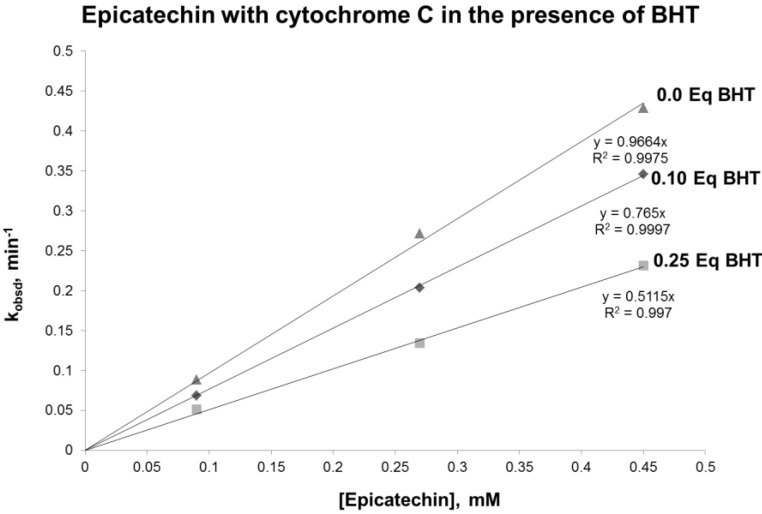
Plot of pseudo-first order rate constant, k_obsd_, *vs.* the concentration of epicatechin in the presence of 0.0.1 and 0.25 equivalents of BHT (butylate hydroxytoluene).

In the proposed mechanism, it is hypothesized that a radical catechin intermediate is formed. To test this hypothesis, the reaction was performed in the presence of BHT, a known free radical scavenger. The results obtained demonstrated a reduced reaction rate when BHT was added to the reaction (Figure 5). Interestingly, the reduction of the rate was proportional to the concentration of BHT. These results support the existence of a radical intermediate.

## 4. Conclusions

Green tea catechins can reduce cytochrome c. The reaction rate of the catechins was dependent on the pH and the nature of the catechin. The pseudo-first order rate constant obtained increases in the order of EC < ECG < EGC < EGCG, which is consistent with previously reported superoxide reduction activities and the Cu^2+^ reduction activities of tea catechins.
